# Performance Assessment for a Guided Wave-Based SHM System Applied to a Stiffened Composite Structure

**DOI:** 10.3390/s22197529

**Published:** 2022-10-04

**Authors:** Inka Mueller, Vittorio Memmolo, Kilian Tschöke, Maria Moix-Bonet, Kathrin Möllenhoff, Mikhail Golub, Ramanan Sridaran Venkat, Yevgeniya Lugovtsova, Artem Eremin, Jochen Moll

**Affiliations:** 1 Institute for Mechanics, Bochum University of Applied Sciences, Am Hochschulcampus 1, 44801 Bochum, Germany; 2 Aerospace Structures and Materials Laboratory, Department of Industrial Engineering, Università degli Studi di Napoli Federico II, Via Claudio 21, 80125 Naples, Italy; 3 Fraunhofer Institute for Ceramic Technologies and Systems IKTS, Maria-Reiche-Str. 2, 01109 Dresden, Germany; 4 Institute of Composite Structures and Adaptive Systems, German Aerospace Center (DLR), 38108 Braunschweig, Germany; 5 Mathematical Institute, Heinrich Heine University Düsseldorf, 40225 Düsseldorf, Germany; 6 Institute for Mechanics, Mathematics and Computer Sciences, Kuban State University, 350040 Krasnodar, Russia; 7 Department of Non-Destructive Testing & Quality Assurance, Saarland University, 66123 Saarbrücken, Germany; 8 Department of Non-Destructive Testing, Bundesanstalt für Materialforschung und -prüfung, Unter den Eichen 87, 12205 Berlin, Germany; 9 Department of Physics, Goethe University Frankfurt, Max von Laue Str. 1, 60438 Frankfurt am Main, Germany

**Keywords:** reliability assessment, guided ultrasounic waves, structural health monitoring (SHM), probability of detection (POD), path-based analysis

## Abstract

To assess the ability of structural health monitoring (SHM) systems, a variety of prerequisites and contributing factors have to be taken into account. Within this publication, this variety is analyzed for actively introduced guided wave-based SHM systems. For these systems, it is not possible to analyze their performance without taking into account their structure and their applied system parameters. Therefore, interdependencies of performance assessment are displayed in an SHM pyramid based on the structure and its monitoring requirements. Factors influencing the quality, capability and reliability of the monitoring system are given and put into relation with state-of-the-art performance analysis in a non-destructive evaluation. While some aspects are similar and can be treated in similar ways, others, such as location, environmental condition and structural dependency, demand novel solutions. Using an open-access data set from the *Open Guided Waves* platform, a detailed method description and analysis of path-based performance assessment is presented.The adopted approach clearly begs the question about the decision framework, as the threshold affects the reliability of the system. In addition, the findings show the effect of the propagation path according to the damage position. Indeed, the distance of damage directly affects the system performance. Otherwise, the propagation direction does not alter the potentiality of the detection approach despite the anisotropy of composites. Nonetheless, the finite waveguide makes it necessary to look at the whole paths, as singular phenomena associated with the reflections may appear. Numerical investigation helps to clarify the centrality of wave mechanics and the necessity to take sensor position into account as an influencing factor. Starting from the findings achieved, all the issues are discussed, and potential future steps are outlined.

## 1. Introduction

Guided ultrasonic waves (GW) have a variety of interesting properties for structural health monitoring (SHM) applications. As elastic waves that propagate in thin-walled structures, they can reach long distances and interact sensitively with different forms of damage, such as cracks, delaminations or even corrosion damage [1,2]. Despite these intriguing features, they have so far not achieved widespread industrial acceptance as a continuous monitoring technique. A key aspect of this is that there is a lack of strategies for performance assessment that take into account the peculiarities of GW-based SHM [3]. In this context, this article addresses the performance assessment of such systems in terms of the probability of detection (POD), looking for the first time into some parameters strongly affecting the reliability. To this end, a case study using using freely available benchmark data from the *Open Guided Waves* (OGW) platform [4] is conducted in order to establish a first proof of concept clarifying the main issues connected to the reliability assessment.

As a starting point, it is worthwhile to look at the determination of POD in the context of conventional non-destructive evaluation (NDE). In NDE, the performance of a method is valid for a defined procedure and a class of structures [5]. This is possible as the measurement device is moved over the surface and the measurements are usually interpreted by the human operators. In the case of SHM, the standard of quantifying the performance of an SHM system is the proof of concept on the structure to be monitored. In simple words, this is done by installing the SHM system on the target structure, taking baseline data, damaging the structure, retaking data and analyzing them with a high degree of automation using computerized methods. This strategy, obviously, is not feasible for highly expensive components, for example, from the aviation industry. This is the reason why numerical tools are often applied in the literature and reversible damage types receive a high attention in experimental studies nowadays [6,7,8,9].

A first example which shows the transition from NDE to SHM was described by Cobb et al. [10]. Here, damage location and damage type were fixed, and standard ultrasonic NDE-transducers based on shear waves were used for the POD assessment. A well-defined damage scenario was used in Janapati et al. [11] showing the results of a combined experiment-based POD supported by numerical simulations. The case study of Aldrin et al. [12] based on fixed damage positions showed a high location dependency of POD. Varying the damage location increases the complexity of POD data acquisition exponentially. Looking at a specific damage location, the experimental data analysis of GW-based SHM sensitivity was addressed by Meeker et al. [13], Kessler et al. [14], introducing the concept of length at detection. This way, the possibility of multiple measurements at one sample is taken into consideration in the statistical analysis. Boller et al. [15] discussed different simulation techniques showing the possible convergence between experiments and simulations for simple structures and structures having features such as stiffeners. In addition, Buethe et al. [16] proposed a model-based POD approach for a composite plate considering the changes caused by changing damage location as an additional influence on the POD. Memmolo et al. [17] determined the POD based on a scalar damage metric and numerical simulations. More recently, Schubert Kabban et al. [18] described how to reduce the experimental effort by combining efficient statistical design and numerical simulations. In addition, Tschöke and Gravenkamp [19] worked on advanced fast simulation methods that can support a model-based POD. Moreover, quantification of detection quality in a way has also been part of many studies aiming at optimal sensor placement. Most define some kind of detection radius for each transducer and focus on finding a full coverage of the structure using the fewest transducers. Flynn and Todd [20] derived a global optimality criterion based on a general formulation of Bayes risk. While the procedure is well derived, it highly depends on a priori assumptions. The focus is on optimal sensor placement and not on quantification of reliability.

Generally, a detailed bibliography on the topic was presented by Falcetelli et al. [21], who focused on the main challenges and barriers that currently prevent the development of proper reliability metrics for SHM. Starting from the analysis of the main differences with respect to POD methodologies for NDE, the authors found that a higher level of statistical expertise is required to achieve POD curves for SHM systems. However, implementing novel approaches for their use is still limited to a few studies in the literature. On the other hand, some simplified experiments have been carried out on extending the reliability concept to complex structures, such as aeronautical composite coupons and subsystems [22]. Falcetelli et al. [21] also oriented the discussion beyond classical POD curves, approaching new metrics such as probability of localization (POL) and probability of sizing (POS) curves. Actually, this aspect reflects the diagnosis paradigm of SHM, which aims to detect, localize and assess the size of a damage. Increasing the level of inspection makes the reliability assessment even harder, and totally new perspectives are needed, as shown by Bayoumi et al. [23] and Moriot et al. [24]. In addition, as the artificial intelligence (AI) algorithms raise interest among the SHM community, another aspect to be considered is the reliability assessment trough the AI approach itself [25].

A performance assessment besides the POD approach can also be realized by a receiver operating characteristic (ROC) curve. Ref. Hong et al. [26] showed uncertainty quantification in guided wave-based testing of a barely visible impact damage in composites. The authors calculated ROC curves based on nonlinear features sensitive to damage. Ref. Liu et al. [27] discussed the calculation of ROC curves for SHM systems based on a combination of experimental and numerically simulated data for pipelines.

While for specific applications with mostly defined damage positions, single approaches on how to assess the performance exist, neither a widely accepted procedure for performance assessment of GW-based SHM systems used for area monitoring of complex structures exists nor does current SHM system development automatically take into consideration the necessity of performance assessment. In addition, there is no attempt in the literature to do this, unless using extensive numerical simulations, such as what was proposed by Gianneo et al. [28], who leveraged MAPOD to derive several conventional POD curves as a function of single parameters such as the flaw size, the angle with respect to the PZT sensors and the Lamb wave mode (A0 or S0). This approach deals with a model-assisted framework aiming at the digitalization of the real structure to study the reliability of the system within the virtual environment [29]. As such, it can provide useful information on the physics of the problem. Nonetheless, it is still challenging to achieve high fidelity in order to estimate how POD is affected by some specific parameters, such as distance from damage, direction of wave propagation, anisotropy and scattering.

The purpose of this publication is to discuss these issues and the underlying interdependencies on a systematic level as well as using exemplary data in a well-established case study. This requires us to review the whole POD procedure and critically assess the effect of any SHM configuration parameter. For this reason, the manuscript is organized in five sections. Section 2 introduces the concept of performance assessment in SHM, describing a variety of relevant factors. Next, Section 3 presents a method for performance assessment based on the POD concept. Section 4 describes the experimental data and damage identification methods used for the case study. Finally, Section 5 includes the case study results and their discussion, focusing on different aspects of the POD calculation. The influence of parameters such as the chosen regression model, the damage position, the threshold for damage detection and the geometrical placement of the path in terms of boundary conditions is addressed. The paper concludes with an overview of the advances made regarding POD and an outline for future work.

## 2. Need for Performance Assessment in SHM

### 2.1. General Remarks on Performance Assessment

**Figure 1:**
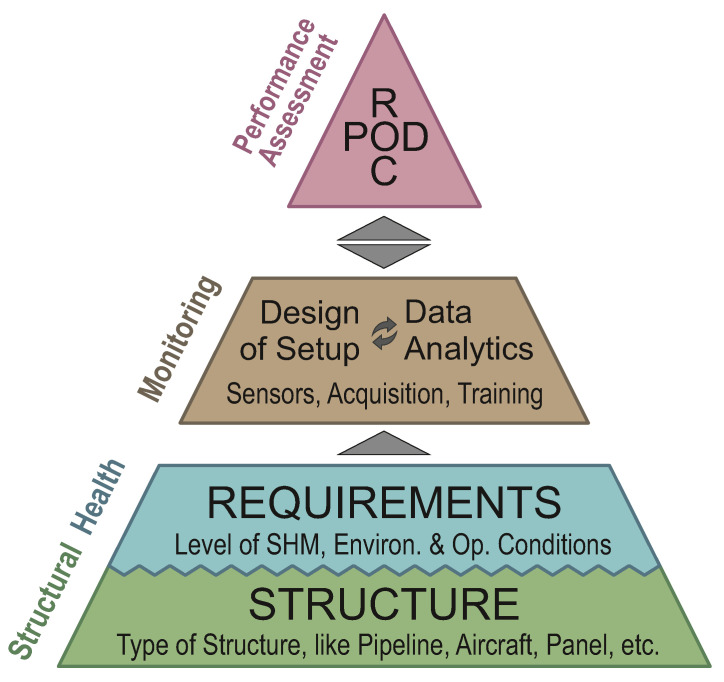
Pyramid of Prerequisites for Performance Assessment of SHM Systems.

Figure 1 illustrates the purpose of performance assessment in the development of an SHM system and its position within the SHM scheme. It is arranged in a pyramid shape to indicate the hierarchical nature of the four-step process: a lower level of the pyramid needs to be fulfilled before dealing with a higher level. The main components of the SHM scheme are the structure to be monitored, the requirements of the SHM system, the damage identification and, finally, the performance assessment.

Every SHM system has a structure as its basis, which has a significant effect on the system. This is especially relevant for structures made of composite materials since these are never the same. Other factors defining the requirements for the SHM system are the targeted SHM level Rytter [30], the damage type and operational and environmental conditions. The structure and requirements define the meaning of the term *structural health*. Any monitoring system must show that it is capable of monitoring this state. Moreover, what defines a monitoring system is not only the type and number of sensors but also the data analysis.

The ultimate goal of SHM is to monitor a health state of a structure using this defined system. However, the performance assessment of the systems must be carried out for its applicability in an industrial setting. If and only if the system is able to match the requirements in a reliable manner, high-quality SHM systems will be sold and used. The development procedure therefore needs to consider performance assessment strategies such as the probability of detection (POD) and/or receiver operating characteristic (ROC) curve.

Both strategies, POD and ROC, consider two parameters of the damage identification: the defect size and the threshold for damage detection. Based on information theory, ROC curves analyze the differentiability between two groups. These two groups are formed by data of the pristine state and data of a specific defect often with a specific damage size. In the ROC, while the defect size *a* is constant, the level of the threshold varies. More information is given in [27,31]. Contrary to ROC curves, in a POD analysis, several defect sizes are considered, while the threshold for damage detection is fixed. In this contribution, the POD is chosen for performance assessment of SHM systems, and further details are given in Section 3.

Only with measurable quantities is it possible to define if requirements are being met. Using these, the monitoring system can be adapted, e.g., updating the data analytics, enlarging the necessary training phase, adopting the sensor positions, etc., until the requirements are fulfilled. It is therefore absolutely necessary to include performance assessment into the general scheme of SHM system development.

Let us compare this general setup to the basic requirements of POD analysis as it is used for quality assessment in ultrasonic NDE. According to [32], a POD analysis must:

1 Define the defect type(s) under study;
2 Define the inspection conditions;
3 Have a sample containing enough realistic defects;
4 Have a suitable technique for validation;
5 Know all relevant defect properties.

While the fourth item is seldom a problem for SHM, all other requirements are connected with difficulties for real-world SHM applications.

Structure and requirements define structural health in SHM. This plateau in Figure 1 is equivalent to the inspection condition, the defect type and the defect properties according to [32]. For quality assessment, the necessary amount and depth of data have to be provided. This is related to the requirement in ultrasonic NDE of being able to have a sample containing enough realistic defects. In SHM, the difficulties of this are manifold, especially as the monitoring system is permanently connected with the structure to be monitored.

For quality assessment of SHM, it is therefore necessary to take into account all of these prerequisites, which not only include the SHM system itself but also the structure as its basis and the assessment method as the peak of the pyramid.

### 2.2. Factors Influencing Performance Assessment

When considering the quality, capability and reliability of an SHM system, four fundamental influencing factors have been identified: *algorithms, intrinsic factors, application factors and integration factors* (see Table 1). Influencing factors have been widely studied in traditional NDE methods [33,34,35,36]. Although some of the factors apply to SHM methods as well, there are some differences to be considered. The main difference between SHM and NDE regarding influencing factors relies on the degree of automatization and of structure-system integration. On the one hand, human and organizational factors represent a major influence in NDE due to manual inspection [37]. On the other hand, the algorithms and intrinsic factors gain importance with SHM due to its high automatization and integration in the monitored structure.

**Intrinsic factors** are inherent attributes of the combined structure-SHM system. Examples of intrinsic factors are the size of damage, level of information needed from the SHM system and the physics of the SHM method, here, guided waves. Intrinsic factors are widely recognized to be fundamental for the SHM system output and its reliability.

**Algorithms** have a major influence on the SHM performance as well. Every decision to develop an SHM methodology influences the end result, starting from the parameters for data acquisition (frequency, mode selection), continuing with the data pre-processing (filtering) and finalizing with the selected damage identification algorithms. With the integration of the monitoring system into the structure, intrinsic factors and algorithms become highly linked.

**Application factors** are influences specific to each application. Examples of application factors are environmental and operational conditions or available information regarding a reference state. Compared to the lab setting, where all application factors can be controlled, the monitoring ability of SHM systems in industrial applications is decreased by the application factors in most cases. It is therefore essential to include these factors in quality assessment strategies.

**Integration factors** are responsible for some of the most significant challenges of performance assessment in SHM and represent major differences between ultrasonic NDE and GW-based SHM. Having a fixed SHM system, the monitoring ability changes significantly depending on sensor positions and damage locations. It is therefore essential to include these factors when evaluating the quality of an SHM system, while at the same time, this is a major challenge for all GW-based systems used for area monitoring, in contrast to hot-spot monitoring.

## 3. Theory of Probability of Detection

The following section provides a brief theoretical background about the POD approach for the reliability assessment of health monitoring systems. The emphasis is posed here upon highlighting critical points and challenges to apply existing methodology to the domain of guided wave-based structural health monitoring. The aim is to prepare a general framework in compliance with regulations and current inspection procedures. Together with the results shown in the next sections, this will pave the way for improving simulation and experiment procedures in order to qualify a method, a system or a methodology for active guided wave SHM.

Despite the fact that many reliability approaches are available, the determination of the probability of detection is the most accepted procedure to quantify the probability to detect a specific defect with an NDE/SHM method. Following this procedure, it is worth achieving the minimum detectable size with a certain degree of confidence, in compliance with the reliability standards. In principle, two different approaches can be used to look into reliability data according to [38]:

- *Hit/miss analysis*, relying on the classic probabilistic approach where binary data are available, i.e., whether or not a flaw is found;
- *$\hat{a}$ vs. a analysis*, based on a mathematical derivation from existing correlation between signal response ($\hat{a}$) and defect size (*a*), if available.

In the former approach, the probability to detect a specific defect is defined as:

(1) $$\mathrm{POD}\left(a^{*}\right)=\frac{n_{h}\left(a^{*}\right)}{N\left(a^{*}\right)}$$

where $a^{*}$ is the nominal dimension of the defect, $n_{h}$ is the number of hit data, namely how many times the system reveals the presence of that specific defect, and *N* is the number of inspections carried out to detect that specific flaw. It is worth noting that the analysis returns a statistically meaningful outcome when statistically independent measurements/inspections are available. Moreover, a physically meaningful result is achieved if all relevant variable parameters are accounted for. Collecting reliability data for a number of flaws with increasing flaw size returns such a binary classification to be interpolated properly. As suggested in [5], a first row manipulation of data consists of building a step function according to the binary findings collected for each specific damage dimension. However, any further classification and performance assessment of the system would not be possible unless thousands of data sets are available. The aim of the reliability procedure is indeed to return the distribution of probability of detection versus flaw size, namely $\mathrm{POD}\left(a\right)$, without making use of countless inspections. In this context, a more affordable possibility consists of estimating a function of the POD versus the damage size matching the values returned by Equation (2) after collection of the binary data. A suited model is described by the cumulative distribution function (CDF):

(2) $$\mathrm{POD}\left(a\right)=1-\frac{1}{1+e^{\alpha +\beta a}}$$

where α and β are coefficients to be computed in order to interpolate the available experimental samples properly. Hence, analyzing binary data indeed requires maximum likelihood estimation to predict the CDF representing the chosen POD model. Furthermore, a hit/miss criterion is needed, such as in the form of a direct threshold criterion, where the detected flaw (outcome higher than the decision level) returns a hit whatever accuracy in flaw size and location is found.

In summary, the hit/miss analysis requires us to:

- Define a decision approach, including how the hit is achieved;
- Define a model function f(a)=POD(a);
- Estimate the model parameters.

**Figure 2:**
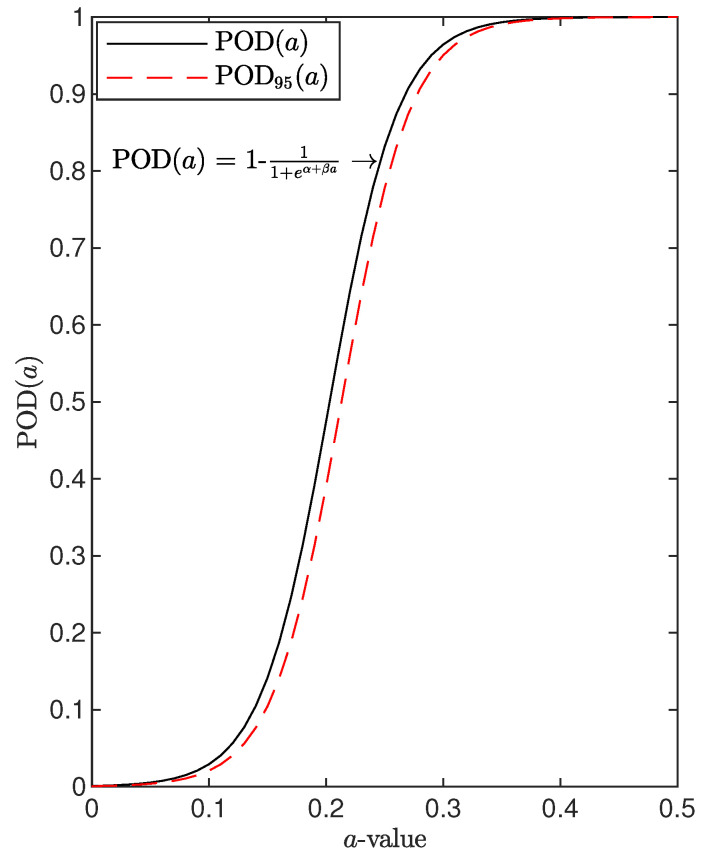
Probability of detection curve versus flaw size, calculated using the hit/miss procedure with the function model and estimation of coefficients.

Figure 2 shows the probability of detection curve versus flaw size obtained applying Equation (2) to artificial data used to demonstrate the concept.

Instead, the latter approach requires us to look into the signal response ($\hat{a}$) and define a model to correlate the POD to a specific flaw (*a*). Even in this case, the probability of detection is defined according to a detection strategy relying on the definition of a decision level (${\hat{a}}_{dec}$), which can be systematically evaluated looking at the noise distribution of the undamaged state. The decision value of the signal response is found by either defining a statistical model assessing the noise of the signal response or through a non-parametric test, both leading to the minimum value of $\hat{a}$ warning actually the occurrence of a damage, which is usually classified as a threshold (${\hat{a}}_{th}$). Approaching an unsupervised method like that presented by [39], it is mandatory to evaluate $\hat{a}$ when no damage at all is indeed affecting the structure. Ideally, the signal response should return a null value. However, due to the inherent noise, a small value is always achieved when returning a certain probability of false alarms. The statistical evaluation of the latter allows us to assess the inherent noise and set the decision level. As to this specific issue, the reader can find a further explanation in Section 5.2.

To proceed with the POD assessment, the first key aspect relies on the assumption that the signal response represents the flaw dimension through a linear relationship. More precisely, such an $\hat{a}\;vs.\;a$ trend needs to be linear, and to this end, a regression model best fitting the data is chosen. Four functions, f(a), are frequently used, using linear, semi-logarithmic, or logarithmic scales:

(3) $$\begin{array}{c} \hat{a}=\beta_{0}+\beta_{1}a+\epsilon ;\\ \ln\hat{a}=\beta_{0}+\beta_{1}a+\epsilon ;\\ \hat{a}=\beta_{0}+\beta_{1}\ln a+\epsilon ;\\ \ln\hat{a}=\beta_{0}+\beta_{1}\ln a+\epsilon ; \end{array}$$

where the error term ϵ is assumed to have homoscedasticity of variance, which does not depend on the dimension of the damage or the flaw size.

**Figure 3:**
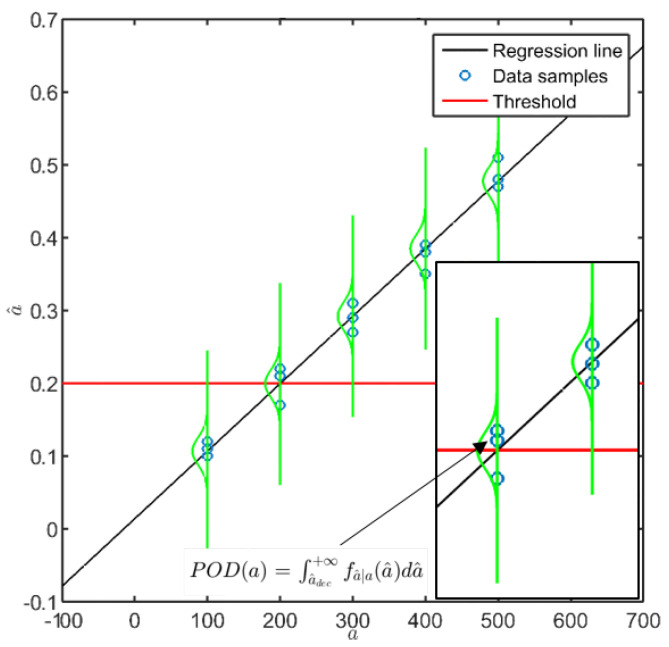
$\hat{a}$ vs. *a* procedure with the regression model and the Gaussian distribution around the predicted value (illustrative data).

The coefficients $\beta_{0}$ and $\beta_{1}$ are to be estimated through an appropriate procedure, for instance using the maximum likelihood approach or ordinary least squares (OLS) regression, where the latter aims to minimize the sum of squared residuals, that is, the difference between observed and fitted values. Once the former coefficients are predicted, the standard deviation around the predicted response is given by:

(4) $$\sigma_{\epsilon }=\sqrt{\frac{\sum_{i=1}^{n}{\left({\hat{a}}_{i}-f\left(a_{i}\right)\right)}^{2}}{n-2}}$$

where *n* is the number of signal response samples. It is worth pointing out that it is a strong assumption that the signal response follows a normal distribution which is moreover independent of the flaw size. From this hypothesis, the signal response belongs to a Gaussian distribution whose mean is the predicted value and standard deviation is $\sigma_{\epsilon }$ (Figure 3).

Hence, the POD can be calculated immediately as the integral above the decision level of the normal probability density function (pdf) around the predicted response:

(5) $$\mathrm{POD}\left(a\right)=\int_{{\hat{a}}_{dec}}^{\infty }f_{\hat{a}|a}\left(\hat{a}\right)\;d\hat{a}$$

According to Berens, the POD function model can be immediately obtained from the CDF of the standard normal distribution:

(6) $$\mathrm{POD}\left(a\right)=\Phi \left(\frac{a-\mu }{\sigma }\right)$$

where μ and σ depend upon the linear regression coefficients and the standard deviation of the predicted value:

(7) $$\mu =\frac{\hat{a}-\beta_{0}}{\beta_{1}};\;\sigma =\frac{\sigma_{\epsilon }}{\beta_{1}}$$

**Figure 4:**
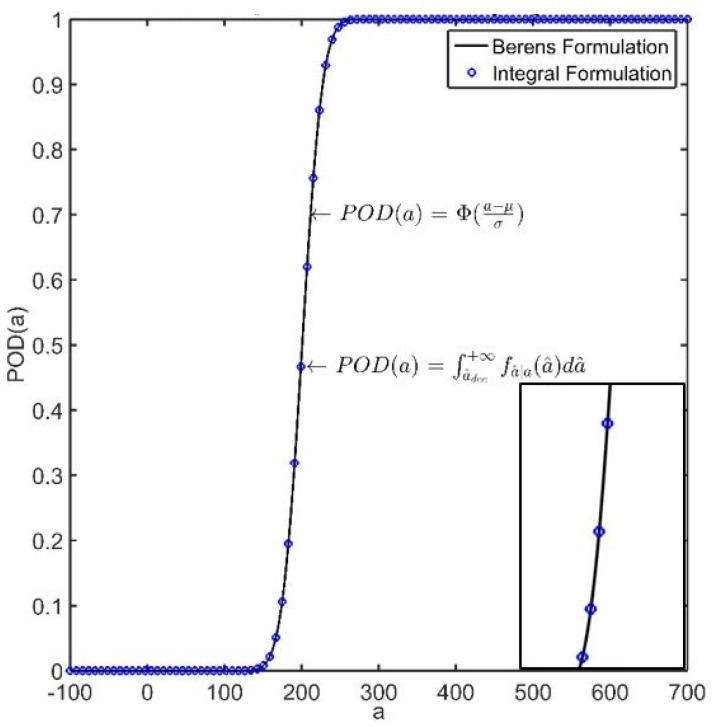
Probability of detection curve versus flaw size, calculated using the $\hat{a}$ vs *a* procedure with the definition of Berens and integral formulation (illustrative data).

The analytical CDF obtained by Equation (7) is depicted in Figure 4 along with the POD calculated by the integral formulation.

**Figure 5:**
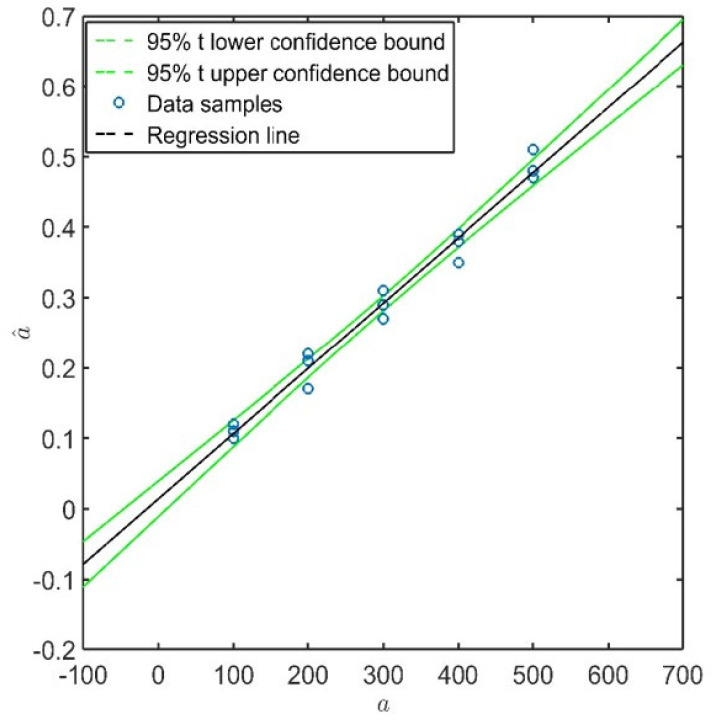
Confidence bounds of the predicted response (illustrative data).

However, the definition of the POD is still variate by way of predicting the signal response through the linear regression. The standard errors of the estimators for the intercept ($\beta_{0}$) and the slope ($\beta_{1}$) are given as:

(8) $$\sigma_{\beta_{0}}=\sigma_{\epsilon }\sqrt{\frac{1}{n}+\frac{\bar{a^{2}}}{\sum_{i=1}^{n}{\left(a_{i}-\bar{a}\right)}^{2}}}$$

(9) $$\sigma_{\beta_{1}}=\frac{\sigma_{\epsilon }}{\sum_{i=1}^{n}{\left(a_{i}-\bar{a}\right)}^{2}}$$

and can be used to derive statistical bounds containing the k−th response with a certain confidence level of 1−α (Figure 5):

(10) $${\hat{a}}_{k}\pm t_{\alpha /2,n-2}\cdot \sigma_{\epsilon }\sqrt{\frac{1}{n}+\frac{{\left(a_{k}-\bar{a}\right)}^{2}}{\sum_{i=1}^{n}{\left(a_{i}-\bar{a}\right)}^{2}}}$$

where $t_{\alpha /2,n-2}$ is the α/2-quantile of Student’s *t*- distribution with n−2 degrees of freedom. According to this definition, if α=0.05, there is a 95% probability that the response falls within these confidence bounds.

The lower confidence bound of 95% is associated with the $POD_{95}$, which returns a 97.5% probability that the effective POD value is actually greater than that. As soon as the $POD_{95}$ is determined, the dimension of the flaw which returns the 90% POD can be considered as the critical damage dimension $a_{90|95}$, which can be detected in a statistically significant way [5]. That is to say, there is 95% confidence in detecting such a flaw with a 90% success ratio.

In summary, $\hat{a}$ vs. *a* analysis requires us to:

- Define a decision level;
- Estimate the regression model best fitting the signal response versus the flaw dimension;
- Select an appropriate approach for obtaining confidence bounds;
- Establish a POD function model.

Using the approach, the signal response of an SHM system as a function of flaw dimension can be estimated, and therefore, the inherent probability of detection can also be estimated. In this contribution, the $a_{90|95}$ value is assumed as the target for the SHM system, thus defining the smallest detectable damage size. Nonetheless, the definition of this target in a guided wave-based multi-input, multi-output SHM approach is still not trivial, as demonstrated hereinafter.

## 4. SHM System

### 4.1. Brief Description of the Data Obtained from the Open Guided Waves Platform

The data set used for the POD calculations is available at the OGW online platform. The data set has been experimentally acquired using a carbon fibre-reinforced polymer (CFRP) specimen with permanently attached piezoelectric transducers. The specimen consists of a flat plate of dimensions 500 × 500 mm^2^ and a 2 mm thickness. An omega stringer of 1.5 mm thickness is bonded at the center of the plate. Guided waves are sent and received by 12 piezoelectric transducers distributed in 2 rows parallel to the omega stringer. Refs. [4,40] present the data acquisition, detailed information on the manufacturing of the plate and the omega stringer, and damage scenarios.

**Figure 6:**
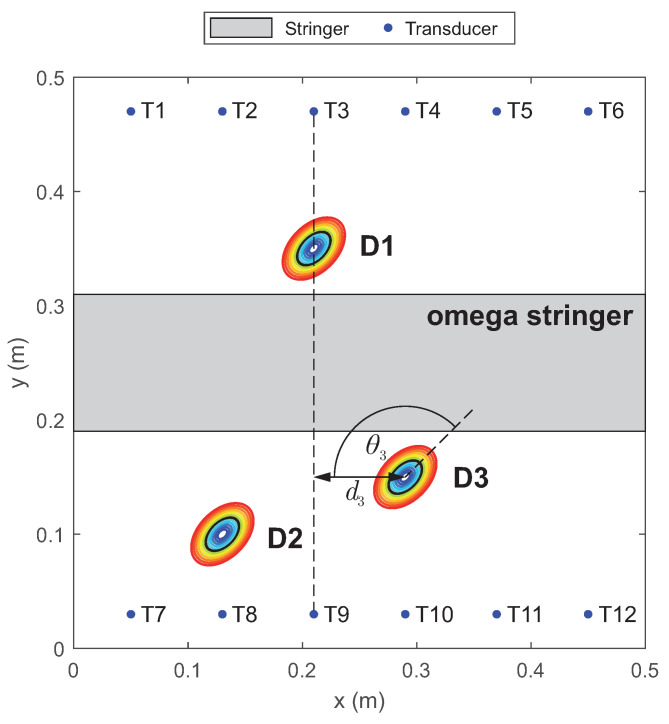
Specimen geometry with the three damage positions D1–D3 relative to the transducer locations T1–T12 ([4]). Considering path T3–T9, $d_{3}$ represents the distance from damage D3, while $\theta_{3}$ is the angle of the transducer pair path against the damage orientation, which is at 45${}^{\circ }$ respect to x-axis.

**Figure 7:**
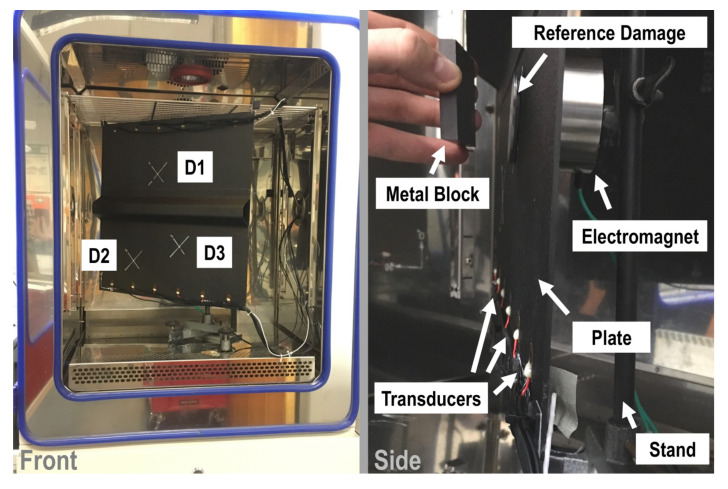
Illustration of the experimental setup arranged in a climatic chamber for the measurement campaign. The plate is instrumented with several transducers and damaged by artificial defect.

Artificial reversible defects of multiple sizes were used to simulate the damage. These reversible defects are metallic plates of elliptical shape which were attached to the specimen at three defined positions. The connection between the defect and the plates was achieved through a vacuum sealant tape based on butyl rubber. The damage sizes ranged from 49.7 mm^2^ up to 2099.3 mm^2^, and 13 damage sized were used. Additionally, the reference damage was de-attached and re-attached five times. This process of de-attaching and re-attaching the reference damage was performed to obtain five measurements as statistically independent as possible, resulting in a populated family of noise data. The specimen geometry, transducer positions and damage locations are sketched in Figure 6. Instead, the experimental setup is depicted in Figure 7.

### 4.2. Damage Identification Procedure

The data set contains the signal response acquired within a frequency range from 40 kHz up to 260 kHz in steps of 20 kHz plus a broadband chirp excitation. Moreover, all possible actuator-sensor pair combinations have been used in a round-robin fashion during the data acquisition. In the following POD calculations, the extensive data set has been only partially used: the complete set of damage scenarios and actuator-sensor pairs were employed, but only a single frequency (40 kHz) was selected. This concentration on one frequency allows us to focus on the POD procedure, while it is explicitly not the focus to find the best frequency for damage detection. Nevertheless, the nature of the damage allows us to assume good interaction with the $A_{0}$ mode, which is dominant in this frequency range.

The $\hat{a}$ vs. *a* approach to POD analysis requires two main inputs: the defect size (*a*) and the defect size estimation derived from the SHM system ($\hat{a}$). While the real damage size *a* is available in the OGW data set, to obtain suitable $\hat{a}$ values, the SHM system must provide a damage indicator that is proportional and sensitive to the damage size. To obtain the damage indicator, the signal response acquired in a pristine state is subtracted from a damaged state. To keep the damage identification method simple, the damage indicator is calculated exclusively at an actuator-sensor pair level. In this work, the focus is on path-based POD analysis, and there is no localization procedure included.

Based on Refs. [4,41], a signal energy-based damage indicator was selected as an input for the POD analysis, which was shown to be sensitive to this type of reversible damage. Considering a specific actuator-sensor pair, the energy difference between two structural states defines the damage indicator DI as follows:

(11) $$DI=\sum_{k=1}^{k=N}{\left(x_{C}\left(k\right)-x_{B}\left(k\right)\right)}^{2}$$

where $x_{B}\left(k\right)$ and $x_{C}\left(k\right)$ are the signal responses of the pristine state and the current state in volts at a time step *k*, respectively. *N* defines the maximum number of data points. In this work, every signal acquired at undamaged state is averaged. A healthy state should deliver a DI equal to zero and increase with growing defect sizes. In the following POD analysis, $\hat{a}$ corresponds to the obtained DI values.

Our focus is explicitly not to find the best suitable damage indicator, but the chosen indicator allows us to focus on the POD analysis. In addition, a single damage occurrence is analyzed, as the goal is to assess the reliability of the system in detecting early damage. The appearance of multiple damages does not change the result qualitatively. Both anomalies would alter the wave propagation, which warns us regarding the damage presence, regardless of its position and number. Instead, this is worth being investigated in assessing damage localization algorithms, which is not part of this investigation.

As explained in Section 2, the integration factors of an SHM system play an important role in its performance assessment and are unique to SHM. One factor considered in this work and described in the next section is the influence of the changing distance between damage and the direct path on the damage identification process. The damage-path distance is defined as the minimum distance between the damage center and the straight line between actuator and sensor; see $d_{3}$ for the line from T3 to T9 Figure 6.

## 5. Results

This section deals with the results obtained by post processing the extensive data set described in the previous section. Since ultrasonic interrogation along many paths is involved, establishing how data processing affects the reliability assessment of the SHM system is not trivial. In addition, the countless possibilities in damage position and orientation exasperates such discussion as it is a matter of combining multiple geometric aspects to obtain all the possible scenarios to predict. To look into the main factors that affect first the way to calculate the POD and then the resulting findings, the following subsections show different separate analysis with increasing depth.

Firstly, the regression models used to get to the specific signal response estimation are investigated to understand the way to comply with the $\hat{a}$ vs. *a* approach. In addition, the effect of regression characteristics exhibited by different paths on the POD is highlighted.

Secondly, the inherent noise of signal response is observed and the possible threshold definition is deeply discussed considering different propagation paths. The aim is indeed to achieve such a rigorous and reasonable decision-making procedure based on unsupervised learning.

Understanding the way to proceed with the POD assessment, the results are discussed according to the multiple inherent nature of GW-based SHM. As a POD calculation along a certain path (single-path POD) is possible, the multiple output needs to be characterized according to geometric factors. As the reliability of the SHM system primarily depends upon how guided waves interact with damage, the effect of the distance between the propagation path and the damage location is discussed to identify any influence on the performance assessment.

To further characterize any possible geometric influence on the performance assessment of the system, how the placement of the damage reflects to the probability of detection is then discussed looking into the geometrical position of paths and their inherent distance and side with respect to the damage. In addition, the angle between the latter and the propagation path is considered as a further possible influencing variable.

Although the reliability assessment is quite well characterized according to the inherent geometry factors, variability is still present in the classification of sensitive paths a priori. To look into this aspect, the selection of the most sensitive paths is finally carried out along with a numerical simulation to show how singular the interaction between guided waves and damage may be and how this aspect can affect the reliability analysis, limiting the generalization of case-by-case findings. It is worth pointing out that the effect of every factor analyzed was never de-coupled by other ones. Otherwise, this section classifies the results according to each of these influencing factors. Indeed, the results are from real measurement campaigns using a distributed sensor network where no effect is mitigated or altered. Each of these effects results in the variability of $a_{90|95}$, and the single factor analysis allows us to evaluate the trends over the specific factor.

### 5.1. Path-Dependent POD Analysis

As aforementioned, one critical step towards the POD assessment relies on the estimation of the regression model best fitting the signal response versus the flaw dimension. This is a critical aspect for the validity of the calculated $a_{90|95}$ value. Different linear models are available as shown in Equation (3). In particular, such an estimation of the model can be established efficiently through ordinary least squares (OLS) approximation, which is by far the most widely used modeling method. As mentioned in the previous section, least squares estimates are given by minimizing the sum of the squared residuals. This way, no additional assumption about the distribution of the samples is necessary. In addition, when the observations come from an exponential family and mild conditions are satisfied, OLS and maximum likelihood estimates are identical, as suggested by [42].

**Figure 8:**
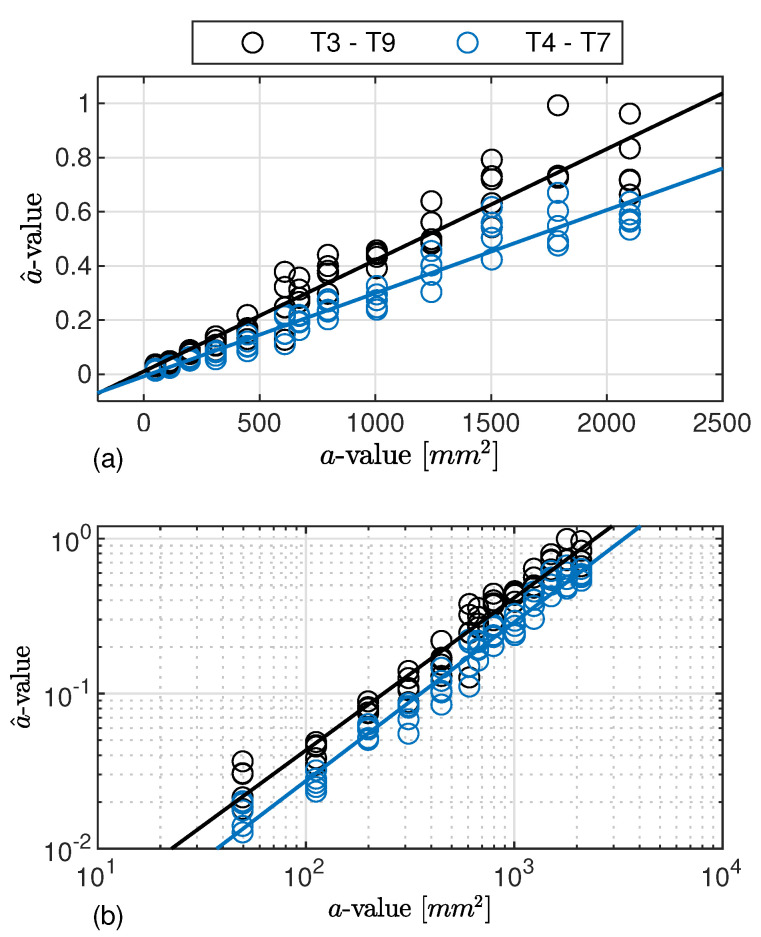
$\hat{a}$ vs. *a* regression analysis. Data samples related to damage D1 and paths T3–T9 and T4–T7. The corresponding linear trend is established using lin−lin (**a**) and log−log (**b**) models.

Accepting linear regression, it is worth starting from the theoretical assumptions of POD to understand that it is essential to get a model tightly falling among the signal response data samples (minimum error ϵ), showing a distribution around the fitting model which keeps constant (same error deviation $\sigma_{\epsilon }\left(\hat{a}\right)$) over the damage dimension and shows a Gaussian behaviour. As to the former aspect, Figure 8 reports the signal response gathered inspecting damage D1 along paths T3–T9 and T4–T7. In particular, among all the linear models, both the lin−lin and the log−log representations show a linear trend of $\hat{a}$ against *a*. Instead, accounting for both semi-logarithmic models, the signal response does not behave linearly at all. This is the reason why their representation is omitted in the plot and the models are neglected in the discussion hereinafter.

Upon a closer look at the path-dependent distribution of signal response versus flaw size, the samples obtained from inspecting the same damage scenario diverge while the latter increases, as seen Figure 8a. Observed samples are indeed tightly located at small values of flaw (very limited error). Meanwhile, a wider distribution of samples around the fitting model emerges as soon as the flaw area increases. That is where the second assumption is indeed not validated because the standard deviation of the error increases with the flaw area. Instead, both assumptions are satisfied, looking into observed values in the logarithmic scale, Figure 8b, whose linear regression law results in the best model fitting the signal response data. It is worth noting that such a distinctive behaviour of lin−lin and log−log estimates stays the same no matter which path is considered.

**Figure 9:**
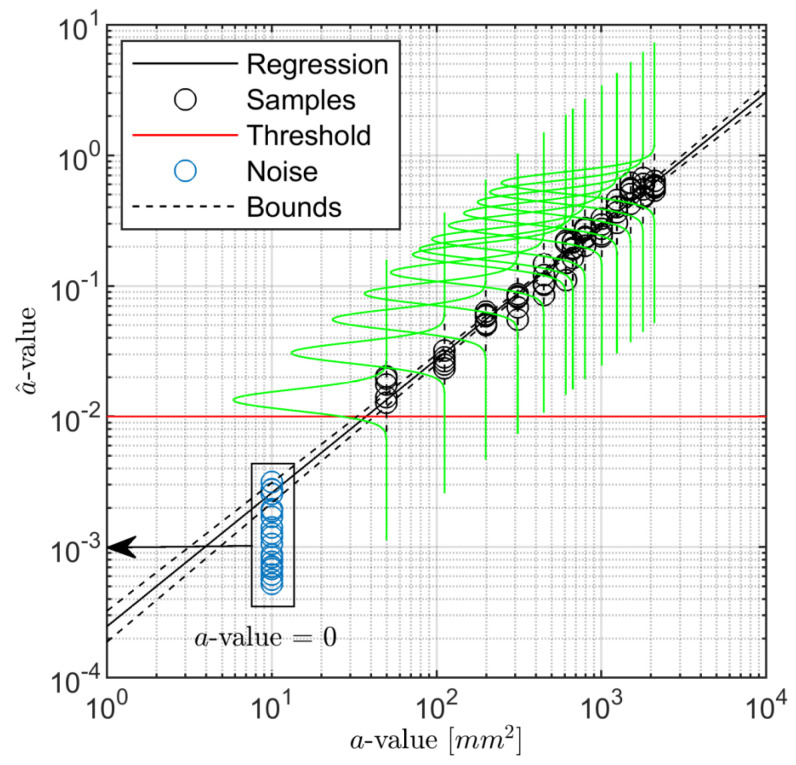
Noise and signal response samples along the path T3–T9 and damage D1. The regression and the signal response distribution are established using log−log model. The *a*-value corresponding to noise samples is zero (no-damage) and is moved along *x*-axis for a better visualization.

To further highlight the suitability of the selected model according to the POD critical assumptions, Figure 9 shows the data samples of path T3–T9 along with the linear estimator and the normal distribution of the predicted response over $\hat{a}$ computed at each considered damage dimension. The regression bounds are depicted in the figure as well in order to have a better impression on how data populate well around the predicted response. The confidence bounds derived in Equation (10) and presented in Figure 5 are very narrow, and thus there may be only a very small deviation in predicting the response by the assessed linear model. Despite the validity in stating that the error of the predicted response is normally distributed, it is worth pointing out that even considering a different distribution family, the procedure remains still valid, and the determination of the POD through integral would return a slightly different value without altering the generality of all the results discussed hereinafter. As to this point, Figure 9 also shows the inherent noise of signal response (replicated and statistical independent measurements carried out when no damage is actually present) and the threshold predicted assuming 90% probability in preventing false alarms. Here it needs to be mentioned that for the majority of paths, the threshold is in the range of measured $\hat{a}$ values, depending on the chosen path and damage location. Further insight about this topic is reported in the following section. Nevertheless, it is worth noting that for the given path, the log−log model returns a signal response, which is quite well distinguishable from the signal noise.

**Figure 10:**
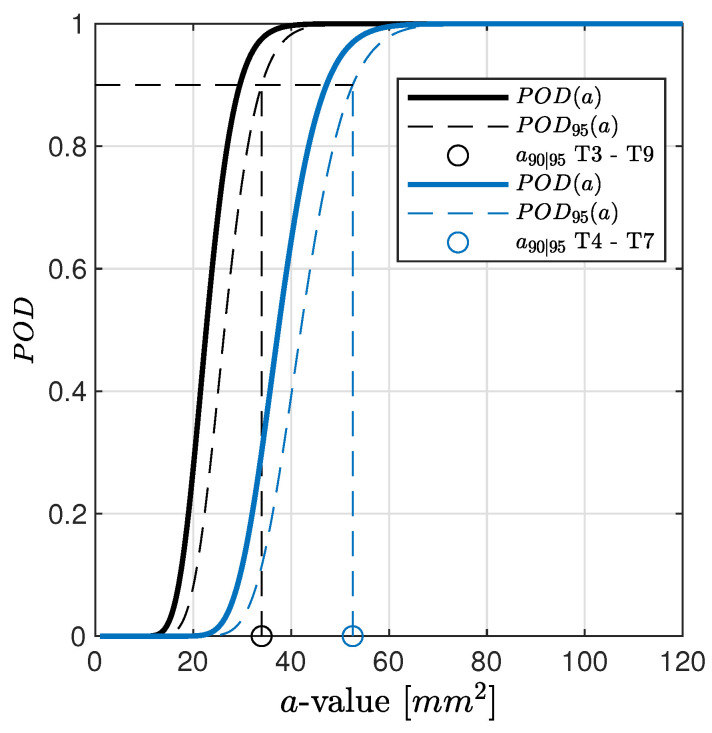
POD versus flaw size related to damage D1 and paths T3–T9 and T4–T7. Regression model used to estimate $\hat{a}$ is according to Figure 8b.

To conclude with this preliminary analysis, Figure 10 depicts the POD assessed considering data samples and the predicted response reported in Figure 8b.

The results show that the estimates such as slope and intercept highly effect the outcome of the path-specific POD curve related to a specific damage location. Knowledge about these effects avoids misleading interpretation. Moving from inspecting the damage D1 along path T3–T9 to path T4–T7, the predicted responses, $\hat{a}$vs.*a*, are different no matter whether they are estimated through a lin−lin or log−log model. In the latter, a slight variation of the intercept value, $\beta_{0}$, is found, while the slope $\beta_{1}$ is almost the same for both paths. This returns a quite visible difference in POD estimation, resulting in an increasing target $a_{90|95}$ with a factor of more than 1.5 (52.58 mm${}^{2}$ vs. 33.97 mm${}^{2}$). This result begs the question of how to address the key aspect of the path-dependent analysis (see the following sections) and consequently remarks the importance of the proper selection of the regression model. That is to say, POD is not only dependent upon the specific path but also on the chosen regression model. The latter influence is where an erroneous or distorted estimator can lead to a misleading assessment of the system performance.

### 5.2. Threshold Dependency

**Figure 11:**
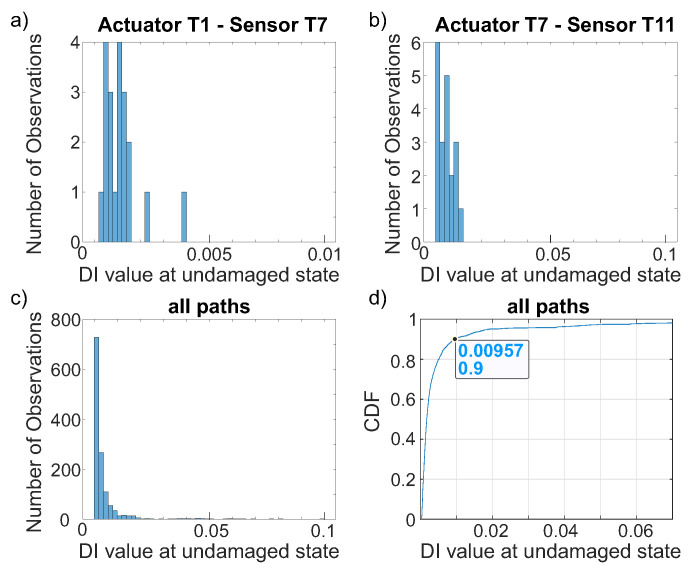
Histogram for damage index values for different paths, (**a**) T1–T7, (**b**) T7–T11, and (**c**) all paths, as well as those that do not cross the stringer. (**d**) Empirical CDF of damage index values given for all paths.

As described in Section 3, the influence of the selected threshold $a_{dec}$ is tremendously important for the calculated $a_{90|95}$ value for a selected path. It is based on agreeing on a defined false call rate. A high false call rate increases the sensitivity but decreases trust in the SHM system. Therefore, it is always a trade-off to choose the false call rate. If the threshold is selected based on an assumed distribution of the damage index values of the undamaged state, it is important to know the type of distribution beforehand. Very commonly, a normal distribution is chosen without proving this. Using Figure 11a, at first an assumption of normal distribution seems to be valid for path T1–T7, but this is not the case for Figure 11b showing path T7–T11. When data of the undamaged state of all paths, also including those paths which do not cross the stringer, are combined (see Figure 11c), the assumption of an extreme value distribution is forced.

Based on this evaluation, extreme value distribution parameters can be estimated to calculate a threshold value $a_{dec}$ based on the chosen false call rate. Alternatively, if enough data are available, it is also possible to work without selecting a distribution model and use the empirical CDF, Figure 11d.

**Figure 12:**
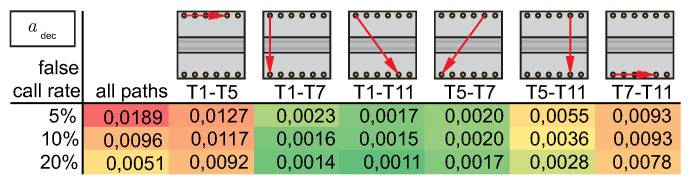
Resulting threshold values $a_{dec}$ based on empirical cumulative distribution of damage index values given for the cumulative empirical cdf of all paths and cdfs of exemplary paths.The fields are colored according to their value; small values are colored green, and high values are colored red.

Taking a closer look at the path-dependent histograms, for the chosen paths T1–T7 and T7–T11, the value range differs by a factor 10, and also, compared to the histogram of all paths, differences are visible; see Figure 11. This is also true for all other paths. A selection of paths is shown in Figure 12. Additionally, values of $a_{dec}$ are given for different false call rates based on empirical CDFs. While paths that cross the stringer (T1–T7, T1–T11, T5–T7, T5–T11) have less variation, it is larger for paths in the upper or lower line of transducers (T1–T5, T7–T11). For the crossing paths, the $a_{dec}$ for a 5% false call rate is smaller than for paths within the line of transducers using a false call rate of 20%, i.e., the influence of path is dominant over the influence of false call rate. Possible reasons are the set up of the structure or a co-influence of the sensor row or sensor electrode orientation. Moreover, some sensors exhibit larger variation within all combinations in this setup, such as T12. Variation in transducers as well as in bonding quality might be a reason for this. The variation in the baseline is again caused by influencing factors, as mentioned in Table 1, emphasising the importance of taking them into account for performance assessment.

Whenever several transducer paths are combined, the final decision on false calls is often made based on the result of several paths. Using the threshold based on all paths, the effective false call rate is increased for some paths, while it is decreased for others. In particular, if only a limited number or baseline measurements is available, this procedure is feasible. If many baseline measurements exist, the alternative procedure of having a path-based threshold can be taken into consideration. As an alternative, it is possible to select groups of paths with similar transducer orientations to improve statistics.

In the following analyses, only those paths that cross the stringer will be analyzed using the same threshold for all paths. The false call rate is set to less than 10%, resulting in a threshold $a_{dec}$ of 0.01. Here, by also taking into account the paths that do not cross the stringer, the selected threshold results in a conservative estimation for all analyzed paths that cross the stringer.

### 5.3. Damage Path Distance Dependency

**Figure 13:**
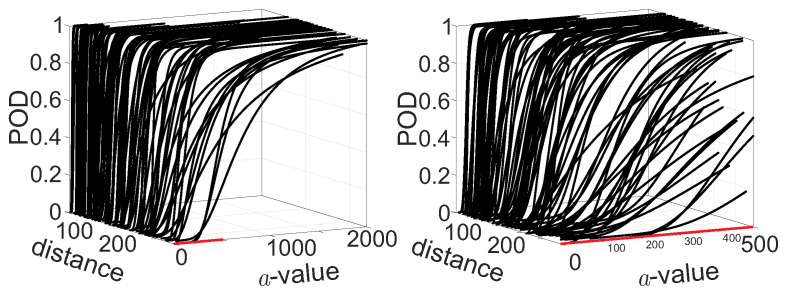
POD curves for all non-horizontal paths, which all cross the stringer over the distance. The right figure shows a detail of the left zooming in to *a*-values from 0 mm${}^{2}$ to 500 mm${}^{2}$.

For all paths crossing the stringer, the POD curves are calculated for all three damage locations using the defined log−log model and a decision threshold of $a_{dec}=0.01$. To test whether there is a significant influence of the absolute distance between the center of the damage and the closest point on the path connecting the actuator and the sensor (see Figure 6 example $d_{3}$), the POD curves are plotted against this distance. It is expected that with increasing distance, the POD curves will shift to higher damage values of *a*, and the slope will decrease. Both effects are visible in Figure 13. Moreover, the zoom on *a*-values between 0 mm${}^{2}$ and 500 mm${}^{2}$ shows that the variation is increasing with increasing distance between the damage center and actuator-sensor paths.

**Figure 14:**
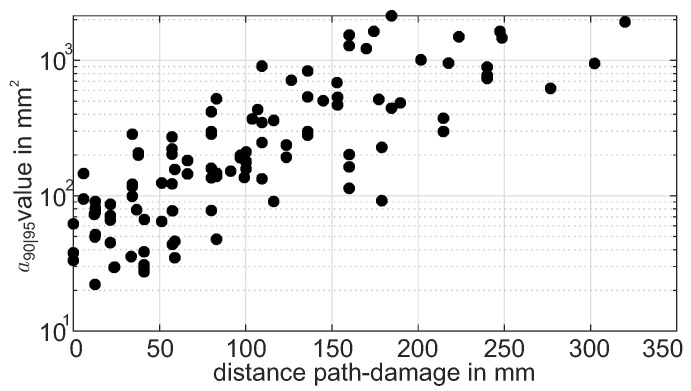
$a_{90|95}$ versus path absolute distance from the flaw as a 2D representation of Figure 13.

To analyze the effect on the $a_{90|95}$ values, the extracted values for all paths crossing the stringer are plotted over the absolute distance from the flaw to the path. The data are shown in a semilogarithmic scale in Figure 14. This way, a good representation of the influence of distance on the $a_{90|95}$ values is given. With the chosen scale, the variation of data is approximately constant over the whole range of distances. For the absolute values, this emphazises the effect of increasing variance with increasing absolute distance values.

For the chosen subset of paths and the selected damage indicator, an exponential dependence of $a_{90|95}$ over the distance between the damage center and path is shown and can be used for further analysis of the performance assessment. At the same time, it needs to be stated that the variation of $a_{90|95}$ at a defined distance is comparably high and non-negligible. The distance is therefore not the only influencing parameter by far.

### 5.4. Dependency on Geometrical Placement of the Paths

The previous section clearly shows that the influence of the path itself in warning the presence of any flaws is dominant on the assessment of the minimum detectable size of damage. An evident motivation relies on the distance of the path from the latter. However, a further reason has to be investigated within the effect of the relative location of the flaw and the transducer pair path (in addition to the absolute distance) with a co-influence of the path against damage orientation.

#### 5.4.1. Effect of Distance Path-Damage

**Figure 15:**
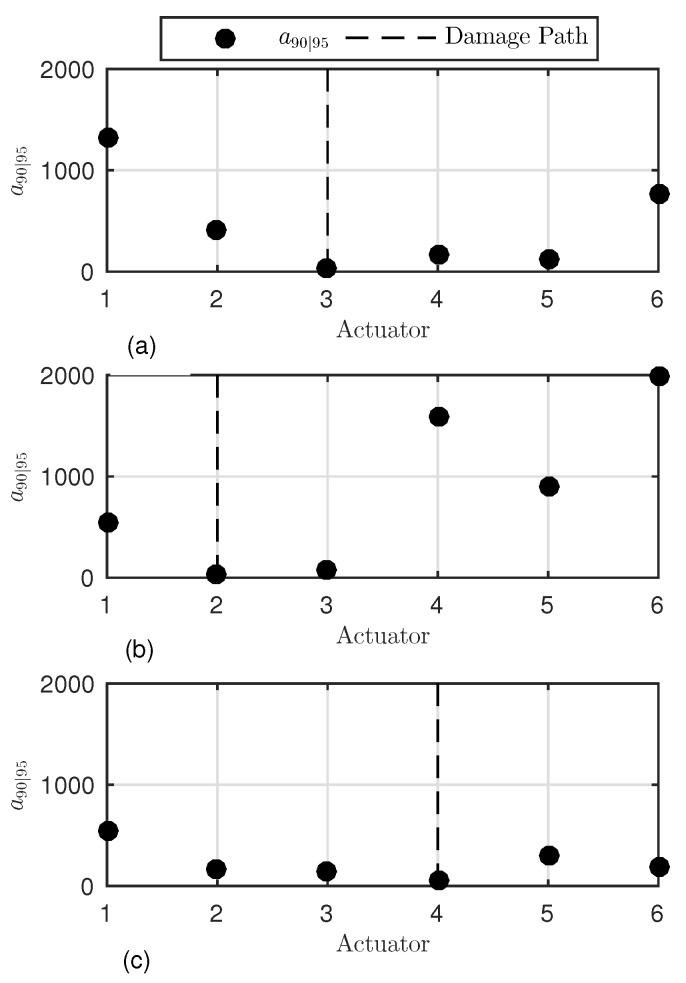
$a_{90|95}$ values versus interrogation path. The POD(a) is predicted along all vertical paths. Damage D1 (**a**), D2 (**b**), and D3 (**c**).

To look further into such spatial dependencies, a first investigation was carried out considering all vertical paths possible, along which the probability of detection was evaluated versus flaw size to assess the target $a_{90|95}$. Figure 15 shows the $a_{90|95}$ values according to the actuator-sensor path for all three different damage scenarios.

These results, visualized for the limited number of actuator-sensor paths, were obtained using the above-mentioned procedure and setting $a_{dec}=0.01$, which corresponds to 90% of the noise CDF. The interrogation path (line of sight between actuator and receiver) is marked by the actuator label and the path crossing the damage is highlighted by the dotted line. Each vertical path is 80 mm distant from the adjacent line of sight, resulting in a different wave interaction with the damage. As highlighted in the previous section, this is a major reason for why a different $a_{90|95}$ is achieved. However, it looks like a non-symmetric behaviour is found around the damage path. The trend of $a_{90|95}$ depends even upon whether the path is located on the left or on the right side of the damage. When damage D1 is considered, the paths on the former side return a lower reliability in detecting damage. Greater $a_{90|95}$ values are found at the same distance from the flaw. Similar results seem to come out from the inspection of damage D2, but the limited number of left-side paths does not allow us to look further into any possible side dependency. When inspecting damage D3, the left-side paths still return slightly different $a_{90|95}$ values on both sides of the damage. Above all else, it is worth noting that the reliability is generally higher and $a_{90|95}$ values are lower than those resulting from the previous cases at the same distance from the flaw.

**Figure 16:**
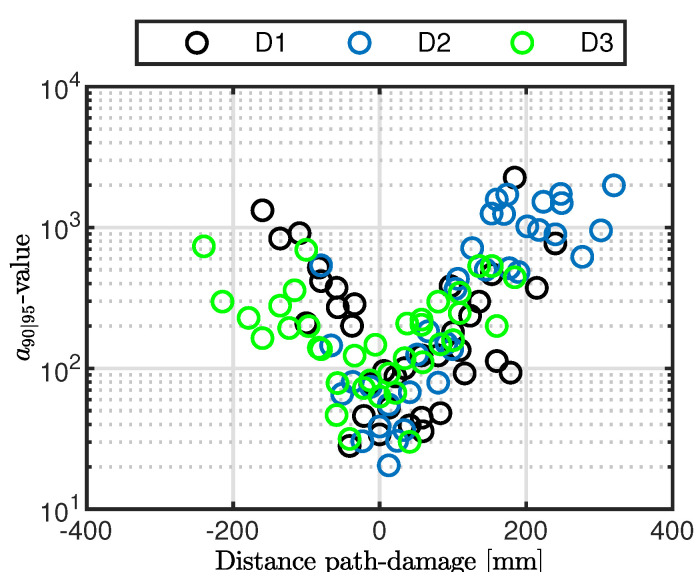
$a_{90|95}$ versus path distance from the flaw. The POD(a) is predicted along all paths crossing the stringer.

However, in all three cases, the outcomes show such an exponential-like trend appearing around the path crossing the damage with a slightly different side lobe distribution. Nonetheless, the lack of symmetry cannot be highlighted due to the limited number of observation samples. Further insight into this aspect is obtained by extending the analysis to all the paths crossing the stringer, which allows increasing the statistics at the cost of introducing another variable dealing with the orientation of the path. The results are plotted in Figure 16, combining the inspection of all three damage scenarios. The different colors adopted allows us to differentiate among several cases. The sign of the distance distinguishes the side of the paths. Those located on the left with respect to the flaw return a negative distance, while interrogation paths on the right side exhibit a positive distance.

When several transducer paths are combined, the lack of symmetry when inspecting a specific flaw is even more evident. However, each damage scenario returns differently populated sides. Only when combining both all the paths and the damage scenarios is the sample family more homogeneously populated over the distance path-damage. In this case, the data show once again a path placement effect. The different distribution of side lobes is characterized by the right paths returning a slightly lower reliability. In addition, the greater the distance, the sparser the populated area, suggesting a larger deviation of the statistics. Far from the damage, the signal-to-noise ratio of the response $\hat{a}$ decreases, and the deviation of the estimate error $\sigma_{\epsilon }$ increases, with a direct effect on the POD and the variability of the assessed performance versus distance. In addition, the semi-logarithmic scale adopted shows that a barely linear trend of $a_{90|95}$vs.distance is found on both sides. Looking into this direction, a slightly lower reliability is visible on the right side due to the higher slope of the trend. Moreover, a constant (logarithmic) deviation is found over the distance, which is due to the greater performance variability far from the damage.

Taking a closer look at the small values, it is also evident that such an exponential distribution is not perfectly focused around zero. Indeed, the performance response at very low distances is quite constant, showing very close values of $a_{90|95}$. Furthermore, Figure 16 clearly shows that the best performance is not achieved by paths exactly crossing the damage. The lowest $a_{90|95}$ values possible are rather obtained mostly along the paths closely crossing the damage.

#### 5.4.2. Effect of Damage Orientation

**Figure 17:**
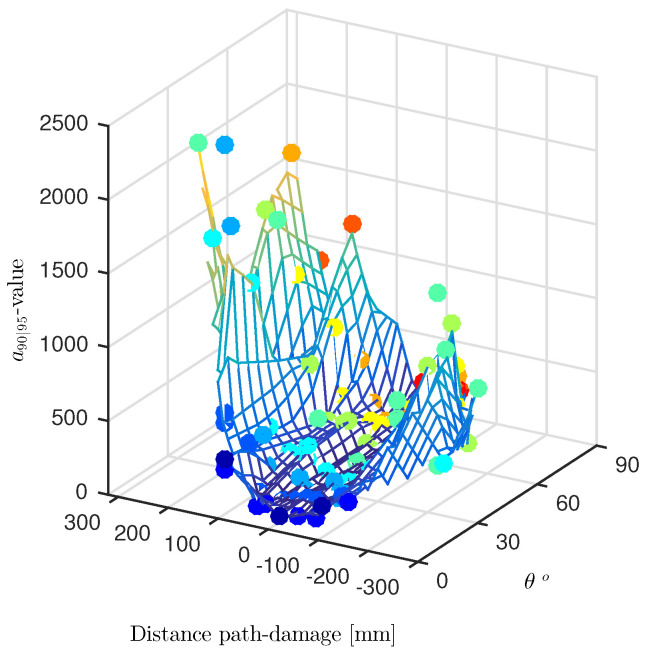
Interpolating surface of $a_{90|95}$ values versus path-damage distance and incidence angle (θ) from the flaw. The POD(a) is predicted along all through the paths crossing the stringer.

Whenever several transducer paths are combined, the distance from the damage can be considered again as a parameter, but the angle of transducer pair path against the damage orientation changes, and it is a further variable candidate. As such, this is accounted for in Figure 17.

Here, the $a_{90|95}$ values are plotted in a 3D diagram over both distance and incident angle θ, defined as the angle between the transducer path and the longer axis of the damage. The scattered data are interpolated through a surface meshed over the distance-angle domain. Both dependencies are marked by a double color scaled representation. Scatter results related to the same θ are indeed highlighted by unique colors, whose scale ranges from red to blue. The former stands for small angles and identifies those paths quite parallel to the damage. Otherwise, the latter stands for greater angles and identifies the paths orthogonal to the flaw. Instead, the surface is color-scaled according to the $a_{90|95}$ estimated by the interpolating model. The hotter the color, greater the value and lower the reliability of the inspection. It is worth pointing out that the scattered data marked with same color belong to a family of parallel paths exhibiting different distances from the flaw.

Having a closer look at all possible dependencies, it is quite clear that the path against damage angle does not show any statistically meaningful influence. Instead, what is more interesting relies once again on the path-damage distance influence. Even reducing the samples by splitting the observation data according to θ, the exponential-like distribution is preserved. In addition, after a first increase, the $a_{90|95}$ values returned by the right paths suddenly drop off to further increases afterwards, showing a local minimum. That is to say, even at higher distances, a good performance of the path-based inspection can be found under certain circumstances.

### 5.5. Discussion of Non-Sensitive Paths

**Figure 18:**
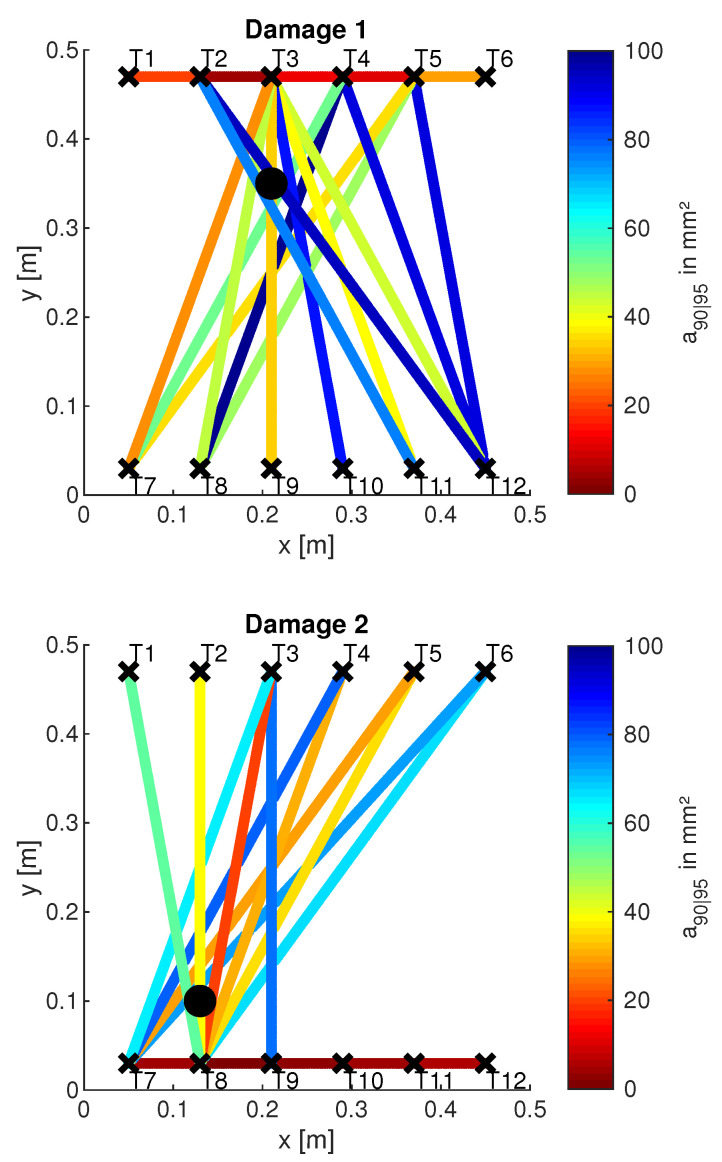

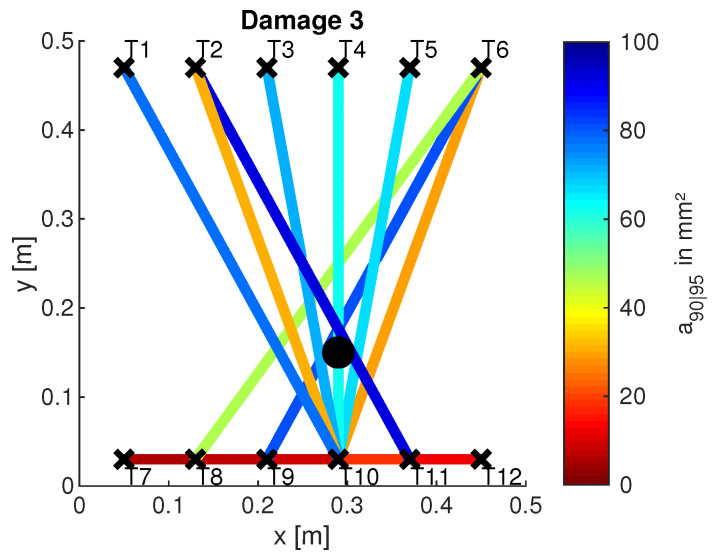
Visualisation of the $a_{90|95}$ values along different paths. A circle ∘ marks the damage position; the crosses × mark transducers. The evaluation was done for $DI_{Energy}$ at f=40 kHz and $a_{dec}=0.01$.

In the previous sections it was shown how POD curves were calculated for selected paths and damage positions. Furthermore, investigations regarding the dependence of the $a_{90|95}$ values on the distance between path and damage as well as on the geometrical placement of the paths have been presented. These results are now summarized for all 3 damage positions and visualized for a large number of actuator-sensor paths. Figure 18 shows the result for $a_{dec}=0.01$. The $a_{90|95}$ is color-coded along different paths, which was determined according to the procedure described above. The color scale ranges from red to blue. Here, red stands for small $a_{90|95}$ values, thus symbolizing sensitive paths. Blue, on the other hand, symbolizes non-sensitive paths. Paths that have not been plotted have an $a_{90|95}$ greater than the maximum indicated in the color bar. They are therefore considered to be particularly non-sensitive.

**Figure 19:**
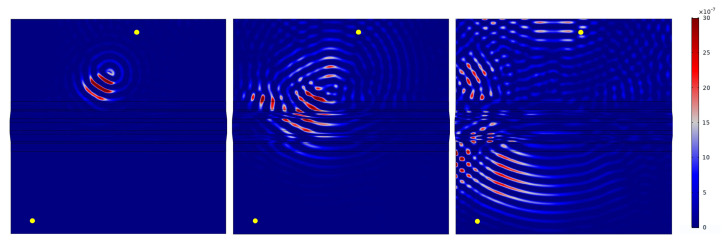
Exemplary representation of the wave propagation in the test specimen. The wave field of the differential signal (undamaged–damaged) is shown for a damage of size 671 mm^2^ at position D1 and the actuator-sensor path T4–T7. The time steps 150 μs, 250 μs and 350 μs were selected.

First of all, it can be noted that the damage can be sensitively detected at every damage location (3 in total) in both transmission and reflection. However, damage can only be detected by reflection measurements if it is in front of the omega stringer from the perspective of the wave front (see also Figure 6). Examples are the paths T2–T3 for damage D1, T7–T8 for damage D2 and T10–T11 for damage D3. In Figure 18, the direct connection between the respective paths is marked in color due to the chosen representation. From the understanding of the wave propagation in the test specimen, however, it is obvious that these results are due to reflections at the respective damage. This statement is supported by the later investigations of the wave fields (see Figure 19). Note that there are both non-sensitive and sensitive paths in the transmission setting that cross the damage. Examples of such sensitive paths are T4–T7 for damage D1, T3–T8 for damage D2, and T6–T10 for damage D3. Examples of non-sensitive paths are T2–T11 for damage D1, T6–T7 for damage D2 and T6–T9 for damage D3. This fact could also be observed for other measurement frequencies and different damage indices. First of all, it is therefore obvious that the material structure of the test specimen and the characteristics of the wave propagation are responsible for this. This will be investigated in Section 5.6, in which first numerical simulation results will be shown to support the observation mentioned here.

### 5.6. Numerical Analysis

The following results show an example of the influence that the geometry of the test specimen and the arrangement of the sensors can have on the POD evaluations shown above. Thus, they provide an explanation for the sensitive and non-sensitive paths described in Section 5.5. The results shown are based on FEM simulations performed using COMSOL Multiphysics software. Indeed, model-assisted analysis can provide an explanation of the physics behind wave propagation phenomena and aid the reliability assessment procedure [29].

The material parameters and plate geometry were used as provided in [4]. A homogenized material model was chosen to reduce computational costs, avoiding fine meshing in the thickness direction. Using the stiffness matrix

$$C={\left(C_{ij}\right)}_{i,j=1}^{6}$$

of a single ply and the lay-up of ${\left[45,0,-45,90,-45,0,45,90\right]}_{S}$, the rotation over the *z*-axis for the angle θ with

$$\begin{array}{c} q=\left(\begin{array}{cccccc} m^{2} & n^{2} & 0 & 0 & 0 & 2mn\\ n^{2} & m^{2} & 0 & 0 & 0 & -2mn\\ 0 & 0 & 1 & 0 & 0 & 0\\ 0 & 0 & 0 & m & -n & 0\\ 0 & 0 & 0 & n & m & 0\\ -mn & mn & 0 & 0 & 0 & m^{2}-n^{2} \end{array}\right) \end{array}$$

and m=cosθ,n=sinθ, the stiffness matrices $C_{k}^{\prime }$ of the rotated plies k=1,...,16 are determined as

$$C_{ij}^{\prime }=q_{ik}q_{jl}C_{kl}\;.$$

By using the equation

(12) $$\begin{array}{c} C_{avg}=\sum_{k=1}^{16}\frac{h_{k}}{H}C_{k}^{\prime } \end{array}$$

the stiffness matrix of the plate using homogenization is as follows:

$$\begin{array}{c} C_{avg}^{plate}=\left(\begin{array}{cccccc} 56.6 & 20.1 & 5.6 & 0 & 0 & 0\\ 20.1 & 56.6 & 5.6 & 0 & 0 & 0\\ 5.6 & 5.6 & 11.2 & 0 & 0 & 0\\ 0 & 0 & 0 & 3.6 & 0 & 0\\ 0 & 0 & 0 & 0 & 3.6 & 0\\ 0 & 0 & 0 & 0 & 0 & 18.2 \end{array}\right)\;GPa \end{array}$$

whereas the homogenized matrix of the stringer is defined as:

$$\begin{array}{c} C_{avg}^{stringer}=\left(\begin{array}{cccccc} 59.1 & 23.1 & 3.4 & 0 & 0 & 6.8\\ 23.1 & 86.6 & 3.6 & 0 & 0 & 6.8\\ 3.4 & 3.6 & 9.6 & 0 & 0 & 0.1\\ 0 & 0 & 0 & 4.8 & 0.2 & 0\\ 0 & 0 & 0 & 0.2 & 4.4 & 0\\ 6.8 & 6.8 & 0.1 & 0 & 0 & 24.9 \end{array}\right)\;GPa \end{array}$$

Here, *H* is the total thickness, and $h_{i}$ is the ply thickness.

To model the plate and the stringer, the COMSOL Solid Mechanics module was used, whereas the piezoelectric transducers were modelled using the Electrostatics module. For each simulation, only the mentioned transducer pair was modeled. The direct solver Mumps was used. An automatically generated tetrahedral mesh was applied using quadratic Serendipity elements and quadratic elements for the Solid module and Electrostatics module, respectively. The element size was 3 mm, and the time step was set as 0.1 μs.

In this contribution, the paths T4–T7 and T2–T11 are presented. First, the respective undamaged state was modeled for both cases. Then, the damage with an area of 671 mm^2^ at position D1 was modeled. The damage is to be understood as a material application made of structural steel. In all cases, a tone burst with a center frequency of 40 kHz and a voltage of 35 Vpp was used as the excitation signal.

**Figure 20:**
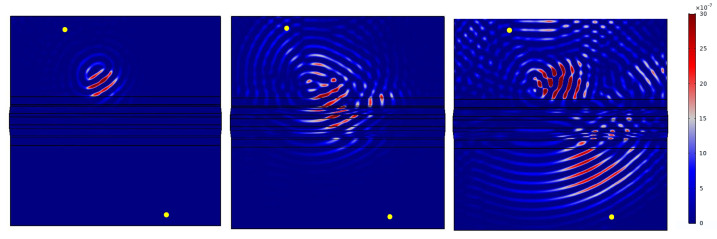
Exemplary representation of the wave propagation in the test specimen. The wave field of the differential signal (undamaged–damaged) is shown for a damage of size 671 mm^2^ at position D1 and the actuator-sensor path T2–T11. The time steps 150 μs, 250 μs and 350 μs were selected.

Figure 19 and Figure 20 show the determined wave fields of the differential signals. That is, for the selected time steps, the magnitude of the displacement resulting from the difference between the undamaged and damaged model is shown. In both cases it can be seen very clearly that the propagation of the differential signal is directional and propagates along the respective path. Similarly, the reflection of the differential signal at the stringer can be seen in both cases. This observation initially supports the results of the horizontal sensitive paths in Section 5.5. Moreover, in Figure 19, it can be seen at 350 μs that the wave is reflected at the left edge of the plate which is then received by the transducer T7. Such a reflection is not observed at the right edge of Figure 20. Therefore, the corresponding differential signal for path T2–T11 does not contain this further reflected signal part.

**Figure 21:**
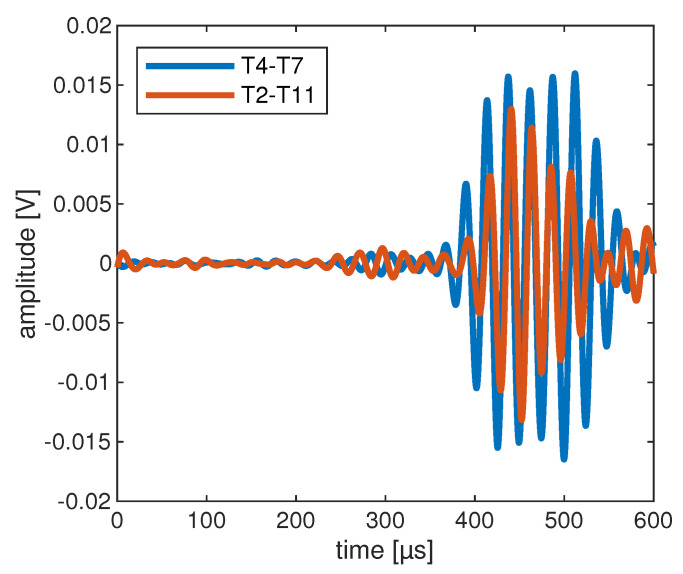
Exemplary differential signals of the OGW data set for a damage of size 671 mm^2^ at position D1 and the mentioned actuator-sensor paths.

Finally, the knowledge gained from the modeling can be used to interpret the signals of the lab measurements as well. As an example, the differential signals of the OGW data set for the paths T4–T7 and T2–T11 are shown in Figure 21. The figure shows the result for measurements with damage at position D1 and a size of 671 mm^2^. In both cases, the averaged signals calculated from the 5 available measurements per path and per damage have been determined (see Section 4). Moreover, the signals were band-pass filtered between 30 kHz and 50 kHz. It can be seen that the differential signal for path T4–T7 is generally stronger than for path T2–T11. Furthermore, the differential signal for the path T4–T7 shows the additional signal component at around 500μs, which results from the reflection at the left edge of the plate. This has already been explained in the description of the wave fields. This signal part coming from the reflection explains the higher damage indicator for path T4–T7 compared to path T2–T11 and thus finally the higher sensitivity of path T4–T7.

The presented modeling results show very clearly how simulations support the understanding of the data evaluation of lab measurements. The influences of the geometry of the test specimen and the arrangement of the transducers can be interpreted only by evaluating the wave fields. This again helps in understanding the lab measurement data and the reasons for the results of the POD investigations.

## 6. Conclusions

POD is one potential solution to the need for performance assessment in SHM systems. When applying the POD concept to GW-based SHM systems, several questions arise. The strong interdependence of performance assessment with the process of SHM system development was carved out in the first part of the paper, introducing a pyramid for performance assessment in SHM. As factors influencing performance assessment, intrinsic factors, application factors, integration factors as well as algorithms have been accounted for. Based on the experimental data set available on the OGW platform, Section 5 gave practical answers to those arising questions, pointing out the influencing factors and the aspects of the pyramid. The main points of the analysis revolved around the regression models used, the choice of a threshold, the damage-path distance dependency and the influence of the geometrical placement of the path.

The first analysis dealt with the regression models based on the least squares estimator to fit the trend of $\hat{a}$ against *a*. While lin−lin and log−log showed linear trends, the independency of the distribution around the regression line over *a* favored the log−log model for all actuator-sensor paths considered. Regarding the predicted response of $\hat{a}$ at each considered damage dimension, the observed data fell clearly within a normal distribution. Thus, a normal distribution was assumed.

The second analysis focused on calculating the threshold. Based on the experimental data, an extreme value distribution could be assumed. Here, alternatively, the empirical cumulative distribution was used as an adequate approach as enough data were available.

Following this, the analysis of damage-path distance dependency showed an exponential dependence of $a_{90|95}$ over the distance between the damage center and the path. The confirmation of this trend is valuable for further analysis of the performance assessment. The last analysis looked into further spatial dependencies with the influence of the path location within a structure using numerical analysis. Independently of the path location, the $a_{90|95}$ values followed an exponential-like distribution when considering the distance from the flaw.

Although the paper looked specifically into many aspects affecting path-based POD, a variety of additional data analyses on other damage identification parameters to be tested in combination of a POD analysis exist. Firstly, different frequency of actuation and damage indices should be analyzed. Secondly, data from all paths should be conveyed to a single information to compress POD outcomes (e.g., this is even more important for localization algorithms) and different kinds of information normalized to obtain unique synthetic data. Thirdly, the influence of distance and POD can be exploited to achieve a map of POD rather than a bench of path-based information. In this view, POD can become an effective tool to assess and optimize the parameters for a certain damage identification algorithm/process.

## Figures and Tables

**Table 1:** Factors influencing the quality, capability and reliability of an SHM system.

Factors	Examples
Intrinsic factors	Physics, SHM level, structure (geometry, material, damage types, critical damage size)
Algorithms	Damage indices, filtering, conventional signal processing or artificial intelligence
Application factors	Baseline, compensation techniques for operational and environmental conditions
Integration factors	Location of damage, location (including integration) of actuators/sensors

## Data Availability

The data presented in this study are available on request from the corresponding author.
